# Tailoring a family-based alcohol intervention for Aboriginal Australians, and the experiences and perceptions of health care providers trained in its delivery

**DOI:** 10.1186/1471-2458-14-322

**Published:** 2014-04-07

**Authors:** Bianca Calabria, Anton Clifford, Miranda Rose, Anthony P Shakeshaft

**Affiliations:** 1 National Drug and Alcohol Research Centre, UNSW Australia, Sydney, Australia; 2 School of Population Health, University of Queensland, Brisbane, Australia; 3 University of Technology, Sydney, Australia

**Keywords:** Indigenous, Aboriginal, Alcohol, Intervention, CRA, CRAFT, Tailor

## Abstract

**Background:**

Aboriginal Australians experience a disproportionately high burden of alcohol-related harm compared to the general Australian population. Alcohol treatment approaches that simultaneously target individuals and families offer considerable potential to reduce these harms if they can be successfully tailored for routine delivery to Aboriginal Australians. The Community Reinforcement Approach (CRA) and Community Reinforcement and Family Training (CRAFT) are two related interventions that are consistent with Aboriginal Australians’ notions of health and wellbeing. This paper aims to describe the process of tailoring CRA and CRAFT for delivery to Aboriginal Australians, explore the perceptions of health care providers participating in the tailoring process, and their experiences of participating in CRA and CRAFT counsellor certification.

**Methods:**

Data sources included notes recorded from eight working group meetings with 22 health care providers of a drug and alcohol treatment agency and Aboriginal Community Controlled Health Service (November 2009-February 2013), and transcripts of semi-structured interviews with seven health care providers participating in CRA and CRAFT counsellor certification (May 2012). Qualitative content analysis was used to categorise working group meeting notes and interview transcripts were into key themes.

**Results:**

Modifying technical language, reducing the number of treatment sessions, and including an option for treatment of clients in groups, were key recommendations by health care providers for improving the feasibility and applicability of delivering CRA and CRAFT to Aboriginal Australians. Health care providers perceived counsellor certification to be beneficial for developing their skills and confidence in delivering CRA and CRAFT, but identified time constraints and competing tasks as key challenges.

**Conclusions:**

The tailoring process resulted in Aboriginal Australian-specific CRA and CRAFT resources. The process also resulted in the training and certification of health care providers in CRA and CRAFT and the establishment of a local training and certification program.

## Background

Aboriginal Australians experience a disproportionately high burden of alcohol-related harm, compared to the general Australian population [1,2]. Approaches that simultaneously target individuals and families at-risk of alcohol-related harm are likely to be acceptable to, and feasible for, delivery to Aboriginal Australians [3], given that positive interaction with family members influences behavioural change in Aboriginal Australians [4], and family relationships are fundamental to the cohesion and wellbeing of Indigenous communities [4,5].

The Community Reinforcement Approach (CRA) [6] is an evidence-based cognitive-behavioural intervention for problem drinkers [7]. CRA aims to reduce alcohol consumption by using social, recreational, family and vocational reinforcers to motivate people towards making their non-drinking lifestyle more rewarding than drinking alcohol. Community Reinforcement and Family Training (CRAFT) [8] is a family-based intervention modelled on CRA and has been identified as an effective cognitive-behavioural intervention with potential to be tailored, in collaboration with locally targeted Aboriginal communities, for delivery in Aboriginal-specific health care settings [3]. CRAFT provides structured, personalised training and support to a family member of a problem drinker. The aims of CRAFT are to teach family members how to effectively and safely remove positive reinforcement for the problem drinker’s drinking behaviour, increase positive reinforcement for non-drinking behaviour, and help to engage the problem drinker into treatment. In addition, CRAFT aims to improve family members’ own social and emotional wellbeing. CRA and CRAFT were both developed in the United States and include a structured certification program for counsellors. CRA and CRAFT are consistent with Aboriginal Australians’ holistic notions of health and wellbeing by incorporating individual and community factors into the treatment approaches [9]. CRA and CRAFT have been found to be acceptable to Aboriginal Australians in rural New South Wales (NSW) for delivery in their local community, when tailored to optimise their cultural appropriateness [10]. Although both interventions have been successfully modified for other minority groups [11-14], they have not been tailored for Aboriginal Australians.

This paper reports on a project that involved researchers working with health care services and Aboriginal community members to tailor CRA and CRAFT for Aboriginal Australians in rural NSW, Australia. The paper has three aims. Firstly, to describe the process of tailoring CRA and CRAFT for delivery to Aboriginal Australians in rural NSW. Second, to explore the perceptions of health care providers participating in the tailoring process. Third, to explore the experiences of health care providers participating in CRA and CRAFT counsellor certification.

## Methods

### Ethics

Ethical approval for this study was provided by the Human Research Ethics Committee, UNSW Australia, and the Aboriginal Health and Medical Research Council, Ethics Committee of NSW. All interview participants provided informed consent.

### Tailoring CRA and CRAFT

**Figure 1:**
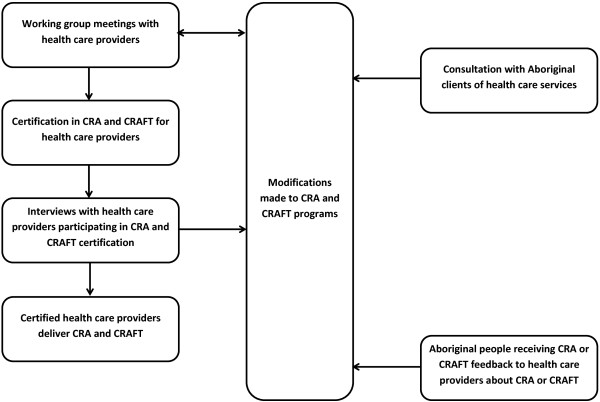
Process of tailoring the CRA and CRAFT interventions.

The process of tailoring CRA and CRAFT for Aboriginal Australians was an iterative process comprising four key phases, as summarised in Figure 1. Each phase yielded qualitative data that informed the tailoring process.

1. Working group meetings with health care providers to adapt CRA and CRAFT for routine delivery to Aboriginal Australians at risk of alcohol-related harm;

2. Survey of Aboriginal clients from participating health care services to examine their perceptions of the acceptability of, and suggestions for tailoring, CRA and CRAFT for delivery in their community [10];

3. Certification of health care providers in CRA and CRAFT to develop their knowledge and skills to deliver CRA and CRAFT in practice; and

4. Interviews with health care providers participating in CRA and CRAFT counsellor certification to explore their experiences of undertaking the counsellor certification programs and perceptions of the feasibility of CRA and CRAFT procedures for delivery to Aboriginal Australians.

This paper presents results from steps 1, 3 and 4. Results from step 2, reported in detail elsewhere [10], are only briefly described in this paper. This method of working in consultation with Aboriginal Australians and health care providers to tailor CRA and CRAFT to be more acceptable to Aboriginal Australians has been previously demonstrated to be feasible in other family-based research with different Aboriginal Australians groups [15]. The process of tailoring CRA and CRAFT used in this project was developed at working group meetings in consultation with Aboriginal and non-Aboriginal health care providers who had local knowledge of the Aboriginal communities and presenting clients, and would likely be delivering the interventions. The development of the tailoring process by researchers and health care providers established a trusting and co-operative partnership, and increased the likelihood that the process would be acceptable to the health care providers who would be participating in it.

The CRA and CRAFT counsellor certification programs were delivered to health care providers by Robert J. Meyers and Associates, internationally recognised trainers in CRA and CRAFT. To complete CRA counsellor certification, participants were required to attend a two day training workshop, demonstrate competence in the 12 core CRA procedures through audio-taped role play therapeutic sessions assessed by a certified CRA supervisor, and participate in a minimum of four supervision meetings facilitated by a certified CRA supervisor. To complete CRAFT counsellor certification, participants were required to attend a two and a half day training workshop, demonstrate competence in the 10 core CRAFT procedures through audio-taped role play therapeutic sessions assessed by a certified CRAFT supervisor, and participate in a minimum of four supervision meetings facilitated by a certified CRAFT supervisor. When certification in one intervention approach (CRA or CRAFT) was achieved, certification in the other approach only required attending the relevant training days and demonstrating competence in four core procedures that differ between CRA and CRAFT. To be certified as a CRA or CRAFT supervisor participants were required to complete the relevant counsellor certification, facilitate five relevant supervision sessions, and assess five relevant audio-taped therapeutic sessions. Certification was awarded by the United States founders and facilitators of the certification process.

The tailoring process involved input and feedback from health care providers on: CRA and CRAFT core procedures; printed resource materials used to work through core procedures with clients (including worksheets for goals of counselling, Happiness Scale, Relationship Happiness Scale, perfect relationship, problem solving, session checklist, and functional analysis); their delivery of CRA and CRAFT; and Aboriginal-specific CRA and CRAFT manuals. Aboriginal-specific CRA and CRAFT manuals were developed from CRA and CRAFT clinical practice manuals [6,8]. The tailored manuals summarised and simplified the content of the original manuals and included scenarios relevant to Aboriginal Australians, in order to better contextualise each intervention’s content and procedures. The manuals were designed to be used for training health care providers in CRA and CRAFT and as a resource to support them to deliver both interventions. Both manuals were written by a researcher with more than 20 years of experience working in Aboriginal primary health care (M.R.).

Input and feedback was also sought from health care providers regarding the most appropriate and feasible research methods for examining the implementation of CRA and CRAFT in their setting and evaluating their effectiveness among their client population using valid outcome measures.

### Setting and participants

Study participants were 19 health care providers from a drug and alcohol treatment agency and three health care providers from an Aboriginal Community Controlled Health Service (ACCHS) in rural NSW, Australia (*n* = 22). The drug and alcohol treatment agency is a non-government organisation providing residential and non-residential drug and alcohol treatment for Aboriginal and non-Aboriginal people. The ACCHSs is a community based organisation with a focus on the social, emotional and psychological wellbeing of Aboriginal people.

All 22 participants attended at least one working group meeting and interviews were conducted with seven working group meeting participants who had completed CRAFT counsellor certification and CRA training days at the time of the interviews. Working group meeting participants were Aboriginal and non-Aboriginal and all interview participants were non-Aboriginal. Table 1 summarises the professional role of participants, their level of participation in working group meetings, and the current level of CRA and CRAFT qualifications they had attained.

**Table 1:** Health care providers’ role and qualifications

**ID**	**Health care service role**	**Meetings attended**	**CRAFT qualification level**	**CRA qualification level**
101	Alcohol and drug counsellor	B, C, D, E	Certified counsellor	Completed training days
102	Family drug and alcohol worker	E, G, H	Certified counsellor	Completed training days
103	Acting director of strategy and planning	A, B, C, D, E, F, G, H	Certified counsellor and supervisor	Certified counsellor and supervisor
104	Alcohol and drug counsellor	E, G, H	Certified counsellor	Completed training days
105	Drug and alcohol mental health nurse	E	Certified counsellor	Completed training days
106	Family drug and alcohol worker	D, E, G, H	Certified counsellor	Completed training days
107	Program manager	A, B, C, D, E, G, H	Certified counsellor and supervisor	Certified counsellor and supervisor
108	Tobacco cessation worker	B	None	None
109	Family worker	B, E	Completed training days	None
110	Chief executive officer	B	Completed training days	None
111	Alcohol and drug counsellor	B, C, D	Completed training days	None
112	Alcohol and drug counsellor	C, H	Completed training days	None
113	Alcohol and drug counsellor	C, E, G, H	Certified counsellor	Completed training days
114	Alcohol and drug counsellor	E	Certified counsellor	Completed training days
115	Administration worker	E	None	None
116	Family worker	E	Completed training days	None
117	Alcohol and drug counsellor	G	Certified counsellor	Certified counsellor
118	Mental health outreach worker	G	None	None
119	Mental health trainee	H	None	None
120	Addiction specialist	A, D, H	None	None
121	Business manager	B	None	None
122	Chief executive officer	B	None	None

Note. A = project development; B = research methodology; C = manual modification 1; D = manual modification 2; E = intervention resources and outcome survey modifications; F = CRA and CRAFT training; G = recruitment 1; H = recruitment 2.

### Data collection

#### Working group meetings

Table 2 presents the overall focus of the eight working group meetings. Working group meetings were an average of four and a half hours each (total of approximately 37 hours), were held at the participating rural drug and alcohol treatment agency or ACCHS, and were chaired by researchers (B.C. or A.C.). The aims and content of each meeting was guided by a pre-planned agenda. All working group members were given the opportunity to have input into the meeting agenda. Detailed notes were recorded at each meeting and distributed to working group members for their comment and validation. Notes were revised in response to working group members’ comments, and then examined for themes relating to health care providers perceptions of the suitability of CRA and CRAFT and the type and level of tailoring required to enhance their suitability for delivery to Aboriginal Australians. Themes identified from notes recorded at group meetings A-F (see Table 2) informed the development of a semi-structured interview schedule to be conducted with those participating in the CRA and CRAFT counsellor certification program.

**Table 2:** Focus of working group meetings

	**Working group meeting focus**	**Date**
A	Project development	November 2009
B	Research methodology	January 2010
C	Manual modification 1	November 2010
D	Manual modification 2	February 2011
E	Intervention resources and outcome survey modifications	November 2011
F	CRA and CRAFT training	May 2011
G	Recruitment 1	September 2012
H	Recruitment 2	February 2013

#### Semi structured individual interviews

Semi structured interviews were conducted with seven health care providers who were recently certified in CRAFT and had completed the CRA training days at the time of interviewing. All interview participants were from the drug and alcohol treatment agency and four had at least one university degree. Interviews were conducted over two days in May 2012 in a private room at the drug and alcohol treatment agency. The interview schedule included questions relating to health care providers’ experiences of participating in CRA counsellor training days and CRAFT counsellor certification, perceptions of the certification process, and perceptions of the suitability of CRA and CRAFT for the treatment and management of Aboriginal people at risk of alcohol-related harm. Interviews were conducted by a qualitative researcher with more than 20 years experience working in Aboriginal primary health care (M.R.) and were an average of 39 minutes duration (range = 29 – 50 minutes). Interviews were audio-taped and recordings were transcribed verbatim for analysis.

### Data analysis

Qualitative content analysis was used to systematically categorise text data into categories derived inductively, and to summarise the data qualitatively [16]. Inductively deriving the coding categories was appropriate given interviews were conducted with health care providers about CRA and CRAFT used in an Aboriginal Australians health care setting for the first time. The unit of analysis for the group meetings was the group and the unit of analysis for semi-structured interview data was the individual. The qualitative content analysis followed three steps: 1) *immersion* resulted in listing memos (nine for working group meeting data and 23 for interview data); 2) *reduction* summarised the memos into codes (five for working group meeting data: CRAFT counsellor certification, therapeutic issues, adaptability of CRA and CRAFT for Aboriginal Australians, modifications to outcome measures, and possible referral pathways; and six for the interview data: CRAFT counsellor certification, CRA training, therapeutic issues, organisational support, qualifications of therapists, and adaptability of CRA and CRAFT for Aboriginal Australians); and 3) *interpretation* identified examples from specific working group meeting notes or from individual health care provider interview transcripts relating to each code [16]. Data pertaining to common codes across working group meeting notes and interview transcripts were combined for interpretation. The primary analysis was completed by the first author (B.C.). Segments of data and emergent themes were reviewed by the second author (A.C.). Disagreements between the two authors were resolved by discussion, with insights arising from these discussions used to refine themes.

## Results

### Tailoring CRA and CRAFT with input from Aboriginal Australians and health care providers

#### Aboriginal Australians

A survey of 116 Aboriginal Australians or non-Aboriginal individuals who had an Aboriginal spouse or child found that CRA and CRAFT were acceptable for delivery in their local community by 95% and 90% of respondents, respectively [10]. There was a preference for counsellors who were known and trusted, and with experience working in the local community. This finding supported the project’s focus on working with the local health care services to train and certify their staff who had experience working in the local community. Aboriginal survey participants also expressed a preference for clients completing CRA or CRAFT to receive follow-up support, and a need for CRA/CRAFT programs targeting young people. Consistent with these views of Aboriginal survey participants, follow-up support at 6 weeks post program completion was incorporated into protocols for CRA and CRAFT delivery, and an adolescent version of CRA shown to reduce substance misuse in high risk young people identified [17,18]. The acceptability of the adolescent version of CRA to Aboriginal Australian adolescents should be examined before tailoring the program.

#### Health care providers

Health care providers were generally of the view that CRA and CRAFT could successfully be delivered to Aboriginal Australians if tailored to their needs, preferences and literacy levels. In particular, modifying language and terminology commonly used in the delivery of CRA and CRAFT procedures to make it more appropriate for Aboriginal people was identified by participants in interviews and working group meetings as important. History, low literacy levels and Aboriginal-specific use of the English language were commonly identified as reasons for changing the language to make it more appropriate to Aboriginal people.

We’re going to have to modify (CRA and CRAFT) a bit for…Australia, for the clients…just the wording and that sort of stuff…maybe use some pictures (health care provider 107).

…you’re working with people who live in these worlds of chaos, you’ve really got to look at the language so that everyday people in that country can relate to it. So I think it really needs to be - an Australian version of it really needs to be looked at. The concepts are still the same but again, you need to look at it in a different way (health care provider 102).

…(with Aboriginal people) you really need to look at what’s happened to them in the past, how would you present this…I think it’s all doable with the Aboriginal community, it’s just that the language might just need to be adapted a little bit to suit them a bit better (health care provider 101).

Language was changed in all CRA and CRAFT resources to be more appropriate for Aboriginal Australians. In particular, technical language was changed to words more regularly used by Aboriginal Australians. CRA and CRAFT resources were modified to include more appropriate language, and clearer formatting in large font. For example, “Who is your loved one usually with when drinking/using?” was changed to “Who is your relative usually with when drinking/using?”

Working group meetings and interviews were used to assess health care providers’ perceptions of the usefulness of resources used to deliver core CRA and CRAFT procedures to clients. Overall, health care providers perceived these resources to be useful, in particular, the Happiness Scale, that asks clients to rate health, social, emotional and economic aspects of their life on a ten-point scale (1 = completely unhappy, 10 = completely happy).

To maximise the likelihood of Aboriginal clients attending and completing CRA and CRAFT, it was suggested at working group meetings that the Aboriginal-specific CRA and CRAFT interventions comprise less than eight sessions (the original United States based CRA and CRAFT interventions are twelve sessions each), and be delivered to clients in groups, as well as to individuals, consistent with the standard model of care used by the health care services. Group programs for CRA and for CRAFT were developed by certified CRAFT health care providers that included the same content as the individual programs; however, the individual programs are designed to use the core procedures in an order that is appropriate for each client and the group programs presents the core procedures in a standardised order using a PowerPoint presentation. Client workbook exercises are also included in the group programs to guide participants through intervention procedures. Clients attend six group sessions and are given the opportunity to attend additional individual sessions if required.

Working group meetings with Aboriginal and non-Aboriginal health care providers were used to gain feedback on early drafts of the CRA and CRAFT manuals. Health care providers said that they wanted “…something to take and something to refer to…not too detailed…more of a guideline” (working group meeting C). Modified examples of how to deliver the interventions were included in the manuals and presented CRA and CRAFT in the Australian context which helped health care providers to translate the United States CRA and CRAFT interventions into an Aboriginal Australian based model of care. When the final versions of the manuals were made available to health care providers for comment and review their feedback was generally positive.

…(the manual) made it a lot easier to understand what each component was (health care provider 106).

When the CRA and CRAFT programs are implemented the certified health care providers delivering the programs will gain feedback from participants and make appropriate modifications to the programs, under the guidance of the research team.

### CRAFT counsellor certification

Health care providers initially perceived the CRAFT counsellor certification process as daunting, but having commenced the process perceived training days, audio-taped sessions for review and feedback, and supervision sessions, as valuable for developing their skills and confidence in delivering CRAFT.

(I) enjoyed the CRAFT training, I thought it was really good…(the CRAFT counsellor certification process has) been really, really valuable (health care provider 103).

As a component of CRAFT counsellor certification, fortnightly supervision sessions were delivered to health care providers via videoconferencing using Skype [19] or ooVoo [20]. These sessions were delivered regularly to enable the United States based CRA and CRAFT certified supervisors to meet with health care providers undertaking CRAFT counsellor certification in Australia. The purpose of these meetings was for supervisors to redemonstrate CRAFT procedures to health care providers and provide them with group level feedback on their performance to date. Health care providers reported that supervision sessions were important for developing their confidence and skills to deliver CRAFT procedures to the standard required to complete the certification process, and for addressing their questions or concerns relating to delivering CRAFT in practice.

I thought that (supervision on Skype) was a very useful tool, rather than reading. I think it was good because you could then ask questions directly (health care provider 103).

Although feedback about the supervision sessions was positive, health care providers reported experiencing some technical difficulties when using the Internet-based programs for video conferencing. Two health care providers are now certified CRAFT supervisors so ongoing CRAFT supervision can be delivered face-to-face and will resolve these technical issues.

### CRA training days

The two day CRA training was delivered after the two and a half day CRAFT training. Given that some core CRA and CRAFT procedures are identical, there was some repetition. Nonetheless, the CRA training days were well received by health care providers.

For those of us who had done CRAFT, we found the first day (of CRA training) a little boring…but the second day, we did quite a few more exercises and…some group stuff (health care provider 103).

### Local training and certification program established

In this study, high staff turnover prompted the establishment of a locally based training and certification program run by the two certified CRA and CRAFT supervisors. In addition to training days and reviewing audio-taped sessions, these supervisors have developed a system whereby a certified counsellor delivering CRA or CRAFT group sessions is assisted by a counsellor in training, rather than another certified counsellor. This process ensures new staff are exposed to the delivery of CRA and CRAFT in practice while they are undertaking the certification process, and increases the number of staff available to deliver the interventions.

### Therapeutic issues

There were three main challenges identified by health care providers completing CRAFT counsellor certification. First, staying motivated to complete all the audio-taped sessions in addition to their usual workload was difficult for health care providers, all of whom identified this as a challenge.

…the negatives was the time and the pressure that (counsellor certification) put on you (health care provider 104).

…(the CRAFT counsellor certification process) was challenging…fitting it in with work (health care provider 105).

This challenge was resolved by nominating an individual to co-ordinate the CRA and CRAFT counsellor certification process and by scheduling specific days for health care providers to complete the required audio-taped sessions, without the distraction of competing work tasks.

The second challenge of the CRAFT counsellor certification process was initial reluctance by health care providers to role play therapeutic procedures; however, the benefits of role plays for consolidating and applying knowledge acquired from training days became apparent to health care providers once they participated in role plays, and their reluctance appeared to diminish.

…it was kind of hard to do (role plays) and feel natural about it…the more we did it, the more you got used to that (health care provider 104).

…I am a lot more confident with using (CRAFT) now than I would have been if we hadn’t have done the role plays before and just getting the feedback was good to know this bit you did really well, this bit you probably need to work on (health care provider 106).

The third challenge was the incongruence of some core procedures with health care providers’ previous counselling training. Concern was raised, in interviews and working group meetings, about a process included in CRAFT that asks the family member of the drinker how they think their relative feels about his/her own drinking and what they believe their relative is thinking when he/she drinks. Health care providers generally perceived this question to be inappropriate.

I’d always learnt in counselling that you don’t get people to theorise or hypothesise about how someone else feels (health care provider 103).

…trying to get an understanding of what does your loved one think right before he drinks?, or what does he feel right before he drinks? I think that’s going to be pretty hard for people and I guess how accurate that’s going to be is a bit of a concern I think (health care provider 106).

### The role of organisational support

Health care providers felt well supported by their organisation and identified this support as important. Support and encouragement from managers and other team members increased their motivation to continue with CRA and CRAFT counsellor certification.

It was really good to sort of work in a team that was doing it together… we sort of kept pushing each other along and we needed to keep going with it, because we were all doing it together (health care provider 104).

### Qualifications of counsellors

Although CRA and CRAFT have previously been delivered, in different settings, by individuals who hold a university degree and have completed CRA and CRAFT counsellor certification, health care providers generally held the view that individuals with different levels of qualifications could effectively deliver the interventions once certified in the approaches.

…so long as you had those people skills and sort of the willingness to sort of learn something different, and then to apply it. Sometimes I think there’s some resistance to apply it, maybe from some lack of confidence. But that’s - I think that’s more down to an individual, rather than their level of knowledge and experience…sometimes probably experience is a more useful thing than having the university education (health care provider 104).

One health care provider suggested that the manner in which certified counsellors will deliver intervention procedures is likely to be dependent upon their existing skills.

I think that there’s quite a lot of high level practice required to implement CRAFT in the way that it’s intended…I think people will vary in the way they implement the procedures (health care provider 103).

### Outcome measures

In tailoring CRA and CRAFT for delivery to Aboriginal Australians, the research team proposed reliable and valid clinical alcohol measures that could be used to identify Aboriginal people likely to benefit from CRA and CRAFT and assess the effectiveness of CRA and CRAFT for reducing alcohol-related harms among those identified to be at risk. These outcome measures not only had to be valid and reliable for the identification and assessment of Aboriginal people at risk, but also acceptable to those health care providers who were responsible for administering them to Aboriginal people, and feasible for them to do so in the context of routine practice. Health care providers deemed the package of outcome measures originally proposed by health researchers as too lengthy and time consuming to complete. The package of outcome measures originally proposed aimed to measure drug and alcohol consumption, and social, emotional and physical wellbeing. The package of outcome measures comprised demographic questions, the Emotional Empowerment Scale (EES14) [21], K-5 [22], Growth Empowerment Measure (GEM) scenarios [21], Alcohol Use Disorders Identification Test (AUDIT) [23], frequency of illicit drug use, Assessment of Quality of Life - 6D (AQoL-6D) [24], time spent caring for a problem drinking relative (in CRAFT outcomes only), and health care service use questions. In response to health care providers’ feedback, the package of outcome measures was revised to include demographics questions, the Alcohol, Smoking, and Substance Involvement Screening Test (ASSIST) [25], K-5 [22], GEM scenarios [21], and health care service use questions. The aims of the intervention evaluation were not compromised by changes to the main outcome measures.

### Recruitment options

Working group meetings G and H were used to identify and discuss recruitment options that would have the potential to develop into ongoing referral pathways into CRA and CRAFT once implemented into routine practice. Health care providers suggested a number of options including through probation and parole, and other local health care services that do not currently provide alcohol treatment services for Aboriginal Australians.

## Discussion

CRA and CRAFT were tailored for Aboriginal Australians with input from Aboriginal individuals and health care providers. Modifications to the CRA and CRAFT interventions to improve their appropriateness for Aboriginal people included: simplification of technical language to words more commonly used and understood by Aboriginal Australians, clearer formatting and larger font on intervention resources, inclusion of Aboriginal-specific scenarios in CRA and CRAFT manuals, reduction in the number of individual treatment sessions from 12 to six, the inclusion of group sessions and follow-up support for clients six weeks after their completion of CRA and CRAFT. Counsellors that were know and trusted by clients were trained and certified in CRA and CRAFT.

CRA and CRAFT counsellor certification involved a lengthy process that was positively reviewed by health care providers at participating health care services. Initial challenges of time constraints and reluctance to perform role plays were resolved by organising specific days to complete certification measures and by ongoing organisational and peer support. Underfunding of Aboriginal health care services [26,27] often means that employed health care providers have a large workload, and reiterates the importance of organisations assigning a co-ordinator to ensure health care providers are motivated and given time to participate in activities designed to improve their knowledge and skills to undertake their professional role. Underfunding also affects continuity of staff employment [28]. In this study a locally based training and certification program run by the two certified supervisors helped to train new staff.

CRA and CRAFT counsellor certification were completed in the original United States versions of CRA and CRAFT. The CRA and CRAFT concepts and resources were thought to be applicable to Aboriginal Australians, when tailored specifically for this population group. Changing the contextual focus has been the aim of other research projects tailoring CRA or CRAFT for minority groups [12,14]. For example, alcohol dependent Native Americans were reconnected with their cultural spirituality through cultural practices, such as the talking circle, while participating in CRA [14]. Other Aboriginal-specific interventions have also focused on changing the context of an intervention, such as using story telling with parents and children to improve behavioural problems and decrease parental psychological distress [29,30]. The establishment of a locally based training and certification program run by the two certified CRA and CRAFT supervisors provides ongoing support for counsellors certified in the United States versions of CRA and CRAFT to provide the tailored programs to Aboriginal people.

To ensure that CRA and CRAFT can be delivered in routine practice, Aboriginal-specific group programs for CRA and CRAFT were developed by CRAFT certified health care providers. The group CRA and CRAFT interventions are consistent with other intervention programs for Aboriginal Australians. For example, an Aboriginal-specific family-based intervention targeting parents and children aimed at reducing Aboriginal disadvantage [29,30], and a healthy behaviours program promoted through an Aboriginal men’s group [31].

The variation in qualifications held by health care providers involved in CRA and CRAFT counsellor certification was not perceived to be a barrier for delivery but was believed to impact on how the interventions would be delivered. This finding is consistent with studies exploring the optimum mix of qualifications, experience and commitment required to effectively deliver CRAFT [8]. Two individuals have completed CRAFT supervisor certification, providing the opportunity for ongoing local supervision of certified counsellors to assist in intervention fidelity.

An important indication of the success of the tailoring process reported in this paper is that the tailored CRA and CRAFT programs are now routinely available through the participating drug and alcohol treatment agency by certified counsellors, with their impact on client outcomes able to be assessed using the measurement instrument developed as part of this project. Currently, the research team is working with the drug and alcohol agency to conduct a pilot evaluation of their effectiveness in reducing alcohol-related harms.

### Limitations

The purpose of the interviews with health care providers was to explore their experiences of participating in the process of tailoring CRA and CRAFT for Aboriginal Australians and undertaking CRA and CRAFT counsellor certification. Interviews were conducted with a small number of health care providers from one drug and alcohol agency in NSW; however the sample represents 77% (7/9) of all health care providers who had completed CRAFT certification and CRA training days at the time of interviewing. Aboriginal Australian individuals were involved in the working group meetings but none participated in the interviews, predominantly because they were no longer working at the health care service, unable to participate in the certification process due to competing priorities, or appeared unwilling to participate due to fears and concerns they would not successfully complete the certification process. When health care services who work with Aboriginal Australians become involved in research, changes are often required within that service to accommodate the research process. Researchers must also make changes to accommodate the delivery of interventions in a real world setting, as long as the changes do not compromise the integrity and aims of the research. In this project the participating ACCHS was actively involved in early working group meetings, supported their staff to attend CRAFT training, and nominated two health staff to undertake CRAFT certification. Despite this initial interest and involvement, however, the ACCHS’s level of participation in this project diminished. The main reasons for this identified by management of the ACCHS included time constraints due to competing priorities, a lack of staff to cover the duties of health staff participating in CRAFT certification, and the challenges of receiving supervision and support to complete CRAFT certification from a training supervisor from a different organisation located in a town 100 kilometres away. Despite the diminishing involvement of the ACCHS throughout the life of the project, the process of tailoring CRA and CRAFT for Aboriginal Australians succeeded in building expertise in CRA and CRAFT delivery to Aboriginal Australians with alcohol problems in a defined rural region of NSW, and a local CRA and CRAFT supervision and certification program offering the potential for health care providers in neighbouring areas to be trained in the delivery of CRA and CRAFT. The ACCHS has an ongoing relationship with researchers who continue to provide updates on the project.

Although Aboriginal Australian individuals were involved in the working group meetings, and a survey of Aboriginal clients of the participating health services was conducted as part of the tailoring process, the involvement of Aboriginal people could have been improved by consultations with Aboriginal clients regarding their perceptions of the tailored CRA and CRAFT programs.

The self-report nature of the data mean they are prone to bias, in particular social desirable responding is likely. Social desirable responding is likely to have been minimised by anonymity being assured to all participants, and interviews conducted in a private room [32]. Responses in working group meetings may have been biased by group think, but such data are important for the modification of the CRA and CRAFT interventions. The process of tailoring of CRA and CRAFT involved researchers, health care providers and Aboriginal Australians. This paper describes the tailoring process (aim 1) from the perspective of researchers. Researchers examining the implementation of CRA and CRAFT are likely to have different perspectives on the tailoring process than health care providers responsible for their delivery. Health care providers’ perceptions (aim 2), however, were explored, providing insight into the tailoring process from their perspective. Respondent validation of data collected from health care providers enabled researchers to refine their interpretations of the tailoring process in response to health care providers’ feedback.

## Conclusion

CRA and CRAFT were tailored through an iterative process of which consultation between health researchers and health care providers was a main component. The CRA and CRAFT counsellor certification process was perceived to be useful and informative to learn skills and build confidence to deliver the interventions. The content of CRA and CRAFT were believed to be appropriate for Aboriginal Australians; however core intervention procedures and methods for their delivery required tailoring.

## Competing interests

The authors declare that they have no competing interests.

## Authors’ contributions

BC completed the data analysis and took the lead role in drafting the manuscript. BC and MR collected data for the manuscript. MR, AC, and AS critically revised the manuscript. All authors were involved in developing the concept and have approved the final version of the manuscript.

## Pre-publication history

The pre-publication history for this paper can be accessed here:

http://www.biomedcentral.com/1471-2458/14/322/prepub
